# ROS Generation and Redox Enzyme Activity in the Stigmas of Two Tobacco Plant Lines with Different Seed Productivity Levels

**DOI:** 10.3390/cimb48050432

**Published:** 2026-04-22

**Authors:** Ekaterina N. Baranova, Tatiana Kalashnikova, Oksana Luneva, Anna Podobedova, Ludmila V. Kurenina, Alexander A. Gulevich, Inna A. Chaban, Maria Breygina

**Affiliations:** 1 All-Russia Research Institute of Agricultural Biotechnology, Timiryazevskaya St. 42, 127550 Moscow, Russia; ludmila.kur2208@gmail.com (L.V.K.); a_gulevich@mail.ru (A.A.G.); 2 Department of Plant Physiology, Biological Faculty, Lomonosov Moscow State University, Leninskiye Gory 1-12, 119991 Moscow, Russia17a2004@mail.ru (A.P.); pollen-ions@yandex.ru (M.B.); 3 Department of Biophysics, Biological Faculty, Lomonosov Moscow State University, Leninskiye Gory 1-24, 119991 Moscow, Russia; oluneva@yandex.ru

**Keywords:** plant reproduction, stigma, *Nicotiana tabacum* L., ROS, NO

## Abstract

*Nicotiana tabacum* is a classic model for studying pollination on wet stigma. Reactive oxygen species (ROS) and nitric oxide (NO) production are closely related to stigma fertility and depend on the activity of redox enzymes. This study is devoted to the comparison of two tobacco lines differing in physiological parameters and reproductive success. Samsun is a tobacco variety that is widely used in research due to its low demands; however, the reproductive potential of the variety is quite low. Based on this variety, a new line was obtained, called “Fortune”; the plants are externally similar to the Samsun plants, but are more successful in reproduction. The total production of ROS + NO on the stigmas of the Fortune plants is lower than the Samsun plants, but their ROS production is higher, and the main decrease occurs due to NO. Superoxide dismutase activity differs between the two lines at all stages of stigma development except the fertile stage, while ascorbate peroxidase activity is higher in “Fortune” at all stages. Additional isoforms of ascorbate peroxidase are detected in developing stigmas of the Fortune variety. Presumably due to differences in redox metabolism, Fortune plants produce more seeds, their fruit are larger, and their leaves and flowers are also larger compared to the Samsun plants. In this study, we investigated both redox homeostasis parameters and plant productivity using tobacco as the model plant and suggested that there is a correlation between these groups of parameters, which may be important for breeding highly productive plants.

## 1. Introduction

Recently, increasing attention has been paid to the role of reactive oxygen species (ROS) in the control of vegetative development and plant reproduction [1,2,3,4]. These unique molecules can have opposing effects depending on their quantity, the ratio of their forms, and the sensitivity of the cell [5]. Pollen from various plants is highly sensitive to ROS. For example, low concentrations of hydrogen peroxide stimulate the germination of tobacco and spruce pollen and affect cation transport across the plasma membrane and membrane potential in protoplasts isolated from tobacco, *Pyrus pyrifolia*, and lily pollen [6,7,8,9]. Lily pollen tubes are less sensitive to hydrogen peroxide, but other ROS, superoxide radical, stimulates their growth, and the hydroxyl radical inhibits tube elongation and causes polarity disturbance [10].

The level of ROS in reproductive tissues depends on many factors, from the stage of development to environmental temperature [11,12,13]. Thus, by changing the intensity of ROS formation and removal, as well as regulating their interconversion, pistil tissues can stimulate pollen germination, reject it in case of incompatible pollination, or block pollen tube growth in case of unfavorable weather conditions, which is especially important in connection with global climate change [3,14]. The most studied object in relation to ROS regulation of germination among plants with wet stigma is tobacco. It was shown for the first time that exudate—the liquid secreted by the stigma in which pollen germinates—contains hydrogen peroxide, which affects pollen protoplasts [15]; later, the superoxide anion radical was discovered in exudate of tobacco and other plants [16,17].

A shift in the balance between hydrogen peroxide and O^•^_2_^−^ leads to a decrease in tobacco reproductive success: pollen tubes reach the style more slowly, and the number of seeds in the capsule decreases when the stigma is treated with a superoxide dismutase (SOD) inhibitor [16]. Exposure of stigma exudate to ROS quencher or H_2_O_2_-degrading catalase renders it unable to affect protoplasts isolated from pollen in vitro [15]. Thus, for tobacco, the parameters of redox homeostasis on the stigma and the success of sexual reproduction are directly related. Thus, the introduction of an additional SOD gene increases the rate of pollen germination both in vitro and in vivo, while the introduction of choline oxidase gene suppresses it [18].

Samsun is a popular Turkish tobacco variety that has historically been used in high-quality cigars, cigarette blends, and pipes, and is prized for its rich aroma and flavor. This variety has become widely used in research due to its relatively low demands on growing conditions and the abundance of pollen, on which numerous experiments have been carried out [19,20]. However, as we discovered, the reproductive potential of the variety is quite low. This parameter apparently is not taken into account when breeding tobacco plants, since a small number of tobacco seeds is enough to produce a large volume of biomaterial.

This may be of particular importance in connection with significant climate change in tobacco-growing regions associated with global warming [21]. Damage causing a decrease in productivity and fertility is associated with an increase in osmotic pressure, an increase in oxidative stress, a decrease in the intensity of photosynthesis, and the flow of assimilates [22,23,24]. This causes significant changes in both flower formation [25,26], pollen development, and the subsequent fertilization processes [27,28]. When modulating internal stresses using transgenes responsible for the functioning of enzymes involved in the regulation of oxidative and osmotic reactions, disruption of pollen formation, increased oxalate production, and deformation of anthers and pollen were shown [29,30]. Changes in flower morphology accompanying these processes have also been demonstrated [18].

Based on this variety, a new tobacco line was obtained, which we called “Fortune”; the plants are externally similar to the Samsun line, but are more successful in reproductive terms. Taking into account that, for fruiting, unlike tobacco leaf production, fruit size and seed number are of significant importance, the aim of this study was to analyze the two lines with different reproductive potential for the generation and interconversion of ROS on the stigma. We also attempted to demonstrate that control of the generative sphere, namely flower morphology and pollen quality, could be a useful tool in breeding to overcome the adverse effects of ROS-mediated generative sphere growth damage. With some caution, the results obtained in this study on tobacco can be used to select beneficial properties for breeding in other representatives of the *Solanaceae* family. The choice of such an object was supported by the fact that tobacco plants [31], unlike tomato, pepper, eggplant, and potato, have a stigma large enough for ROS analysis both by microscopic methods and by electron paramagnetic resonance (EPR) spectroscopy, which is the most sensitive method of ROS detection [32].

## 2. Materials and Methods

### 2.1. Plant Cultivation and Pollination

Tobacco plants (*Nicotiana tabacum* L.) of the original Samsun line and the ‘Fortune’ line were bred. The line was bred as a result of a series of crosses between the FeSOD-transgenic Samsun tobacco line [18] and a non-transgenic tobacco plant (manuscript in preparation). All tobacco plants were grown in a climate chamber under controlled conditions (25 °C, 16/8 photoperiod) on vermiculite; plants were watered with a nutrient mixture prepared using Nitsch medium during cultivation [33]. The light flux intensity in the chamber was 75–100 μmol/m^2^∙s. Mature pollen was collected from the same plants as the pistils and stigma exudate. For controlled pollination, a standard pollen sample (1 mg) was applied to the pistil and spread evenly with a spatula. Seed set and fruit size were assessed after 3–4 weeks, when the fruit were fully ripe and dried to a constant weight.

### 2.2. Organ Length Measurements and Assessment of Reproductive Success

Organ length was measured on different mature plant individuals. Mid-leaf leaf length was measured from the base of the petiole to the tip of the leaf blade; flower length was measured from the receptacle to the upper protruding part of the corolla.

The diameter of self-pollinated fruit was used as an indicator of reproductive success. Seed set was assessed by weighing the total mass of seeds removed from one capsule. A linear relationship between total weight and seed count has previously been established for tobacco [16]. Figure 2a,b shows immature fruit that set simultaneously. Fully ripe fruit were used for the measurements.

### 2.3. Protein Extraction and Native Gel Electrophoresis of Stigma Proteins

Fresh stigmas were collected from the flowers and homogenized at 0 °C in a tricine buffer (100 mM, pH 8.0) containing 3 mM MgSO_4_, 3 mM EGTA (Merck KGaA, Darmstadt, Germany, ≥97%), 1 mM DTT (Suzhou Yacoo Science Co., Suzhou, China, ≥99%), and 0.1% protein inhibitor (Protease Inhibitor cocktail, Sigma-Aldrich, St. Louis, MO, USA, ≥98%). Homogenates were centrifuged at 10,000× *g*, 4 °C, for 20 min (Centrifuge MiniSpin plus, Eppendorf, Hamburg, Germany). Supernatants containing 20 μg (15 μg for catalase) of total soluble protein were mixed with sample buffer (200 mM Tris-HCl (MP Biomedicals, Illkirch, France, ultra-pure), pH 6.8) containing 20% glycerol, 100 mM DTT, and 0.4% bromophenol blue (HiMedia, Mumbai, India, analytical grade) and loaded onto 12.5% PAAG (7.6% for catalase) (Beijin Solarbio Science and Technology Co., Beijin, China, grade for electrophoresis). Electrophoresis was performed at 180 V for 2.5 h at 4 °C.

### 2.4. Zymographic Determination of SOD Activity

SOD activity was determined as described previously [16]. Briefly, the gel was washed and soaked in 0.5 mM nitro blue tetrazolium (NBT) (Sigma-Aldrich, USA, ≥98%) in the dark for 30 min, transferred to 50 mM phosphate buffer (pH 7.8) containing 22 μM riboflavin (AppliChem GmbH, Darmstadt, Germany, Pure) and 28 mM TEMED (Thermo Fisher Scientific, Sunnyvale, CA, USA, ≥99%) incubated for 20 min, and then exposed to light.

### 2.5. Zymographic Determination of Catalase Activity

The PAAG gel was washed and then soaked in phosphate-buffered saline (PBS, pH 7.6) containing 4 mM H_2_O_2_ for 20 min. It was then briefly rinsed with distilled water, transferred to 1% (*w*/*v*) KCl_3_ and K_4_[Fe(CN)_6_], and exposed to light.

### 2.6. Zymographic Determination of Ascorbate Peroxidase Activity

The PAAG gel was incubated in 50 mM phosphate buffer (pH 7.0) containing 2 mM sodium ascorbate for 30 min, with the solution was changed every 10 min. The gel was then transferred to the same medium additionally containing 2 mM H_2_O_2_ and incubated for 20 min, washed in 50 mM phosphate buffer (pH 7.0) for 1 min, incubated in 50 mM phosphate buffer (pH 7.8) containing 28 mM TEMED and 1.24 mM NBT, and exposed to light.

A ChemiScope 6200 Touch Chemiluminescence Imaging System (CLINIX, Shanghai, China) was used for gel imaging.

### 2.7. Assessment of ROS and NO Generation

The total ROS + NO levels in the stigmatic exudate were assessed using EPR spectroscopy. 1-hydroxy-2,2,6,6-tetramethyl-4-(trimethylammonium)-piperidinium dichloride (CAT-1H, Noxygen Science Transfer & Diagnostics GmbH, Elzach, Germany, ≥95%) was used as a spin probe to determine the total ROS level [34]. To quantify the signal, the intensity of the central line in the EPR spectra was measured. The spectra were recorded at room temperature (21–22 °C) using RE-1307 X–range spectrometer (Pilot Plant of Scientific Instrumentation, Chernogolovka, Moscow region, Russia) operating at a microwave power of 22 mW and a time constant of 0.1 s. Each characteristic spectrum was the result of 5 signal accumulations. Since the baseline signal of CAT1-H is non-zero, control samples were recorded in each experiment; the characteristic spectra in Figure 3a are shown with the spectra of the blank sample subtracted; the values in the diagram are normalized relative to the corresponding controls.

Total ROS production on the stigma was assessed using 2′,7′-dichlorodihydrofluorescein diacetate staining (Figure 4a). To convert a cell-permeable dye into an extracellular one, we used a previously tested de-esterification reaction. A 5 mM DCFH-DA (Lumiprobe Corp., Hallandale Beach, FL, USA, high purity) solution in ethyl alcohol was de-esterified by diluting 5-fold with 10 mM NaOH and incubated for 1 h at 25 °C. The de-esterified dye was then neutralized by diluting 25-fold with potassium phosphate buffer pH 7.0 [35] to a final concentration 200 μM. The stigmas were cut off, placed on a coverslip, and 2 μL of dye was applied to each stigma. The mixture was incubated in the dark for 10 min. The dye in the ester form was also used for staining, but intracellular fluorescence is weaker and varies at different points on the stigma surface. Since the main generation of ROS on the wet stigma occurs extracellularly [36], an apoplastic staining has been presented. Nitric oxide generation was assessed using 4-amino-5-methylamino-2′-7′-difluorofluorescein diacetate (Figure 4c). For de-esterification, a 5 mM DAF-FM DA (Sigma-Aldrich, USA, ≥98%) solution in dimethyl sulfoxide was diluted 500-fold with distilled water. The resulting solution was diluted 5-fold with 10 mM NaOH and incubated for 1 h at 25 °C. The de-esterified dye was then neutralized by diluting 5-fold with a potassium phosphate buffer at pH 6.0. Plant pistils, excised at the base, were incubated for 10 min in a MES-KOH buffer (pH 6.0) (MES, Macklin, Shanghai, China, ≥99%), and followed by 10 min in the de-esterified dye.

Quantitative and qualitative fluorescence microscopy was performed using an Axioplan 2 imaging MOT widefield fluorescence microscope (Carl-Zeiss-Stiftung, Oberkochen, Germany) equipped with an ADF Pro 08 digital camera and a mercury lamp. A FITC filter set was used for DCFH and DAF-FM.

### 2.8. Statistical Analysis

Experiments were performed on three to eight independent flowers. For each line, four–six plants were continuously in bloom, planted two per pot, three pots per line. All pots were kept under identical conditions, randomized weekly, and labeled. Results in the text and figures, with the exception of original images and representative spectra, are presented as mean values ± standard errors. Significant differences were assessed using the Mann–Whitney test for small samples (* *p* < 0.05).

## 3. Results

### 3.1. Features of Vegetation and Reproduction of the Samsun and Fortune Plants

**Figure 1:**
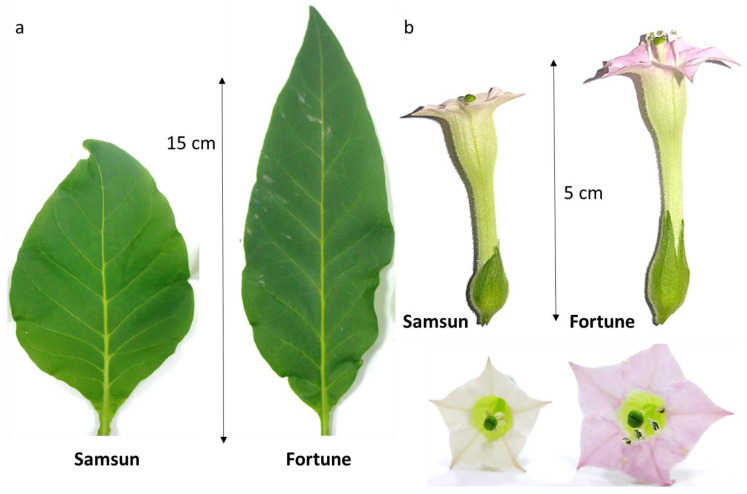
Dimensions of leaves of the middle tier (**a**) and flowers (**b**) of tobacco of the two lines: Samsun and Fortune; as a result of uneven elongation, the relative position of the anthers and stigma differs in the plants of the two lines: in Samsun plants, the stamens do not reach the stigma; in Fortune plants, they are at the same level.

**Figure 2:**
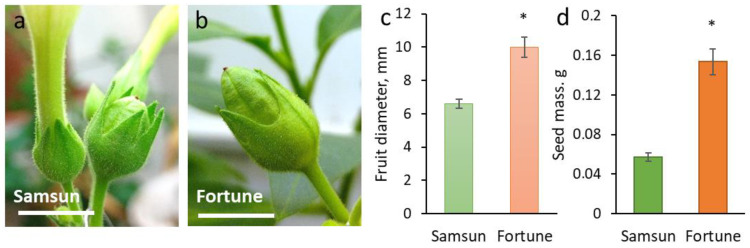
Reproductive success of tobacco plants of the Samsun and Fortune lines, results of pollination of the stigmas with 1 mg of their own pollen: (**a**,**b**)—fruit 12 days after pollination; (**c**)—fruit diameter; (**d**)—total mass of seeds in one capsule; * indicates significant differences between the lines by the Mann–Whitney test (*p* < 0.05).

Visually, the differences between plants of the Samsun variety (S) and the Fortune line (F), were manifested in larger sizes of leaves (Figure 1a), flowers (Figure 1b), and fruit (Figure 2a–c), as well as in an increased number of seeds (Figure 2d) in F plants compared to the original variety. Differences were also detected in the ratio of the lengths of the stamens and pistils (Figure 1b). In F plants, the stamens and pistils are the same length, which promotes self-pollination; in S plants, the stamens are shorter than the pistil, so in the absence of targeted pollination, the pistil remains unpollinated. Controlled pollination with self-pollen (by equalizing the amount of pollen for each flower) resulted in a different seed set, which is reflected in the total seed mass from one capsule (Figure 2).

### 3.2. Generation of ROS and NO by the Stigmas of the Two Tobacco Plant Lines

**Figure 3:**
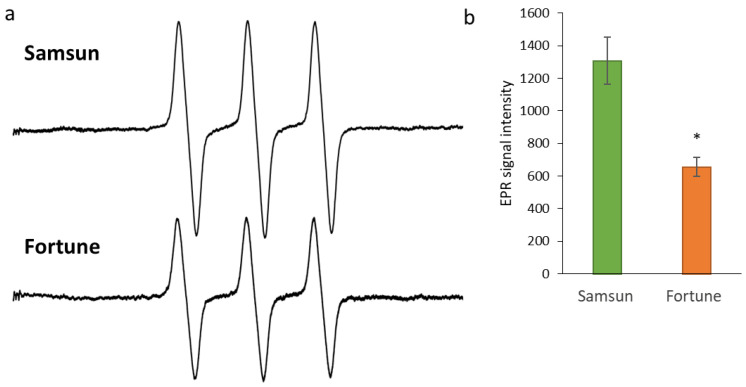
Generation of ROS and NO (total oxidative capacity) on the stigmas of tobacco plants of the Samsun and Fortune lines, assessed using EPR spectroscopy and the CAT-1H spin probe at the stage of maximum fertility: (**a**)—characteristic EPR spectra, (**b**)—quantitative assessment of the EPR signal averaged over several experiments; * indicates significant differences between the lines by the Mann–Whitney test (*p* < 0.05).

The total accumulation of ROS and nitric oxide in the stigma exudate was assessed using EPR spectroscopy with a non-specific spin probe CAT-1H. This sensitive method allows for an assessment of the total oxidizing capacity (OC) of a sample without specifying particular components (ROS and NO). The signal from the stigma exudate of the S plants is significantly greater than that of the F plants (Figure 3a), the average values for several experiments (n = 8) are presented in the diagram (Figure 3b).

**Figure 4:**
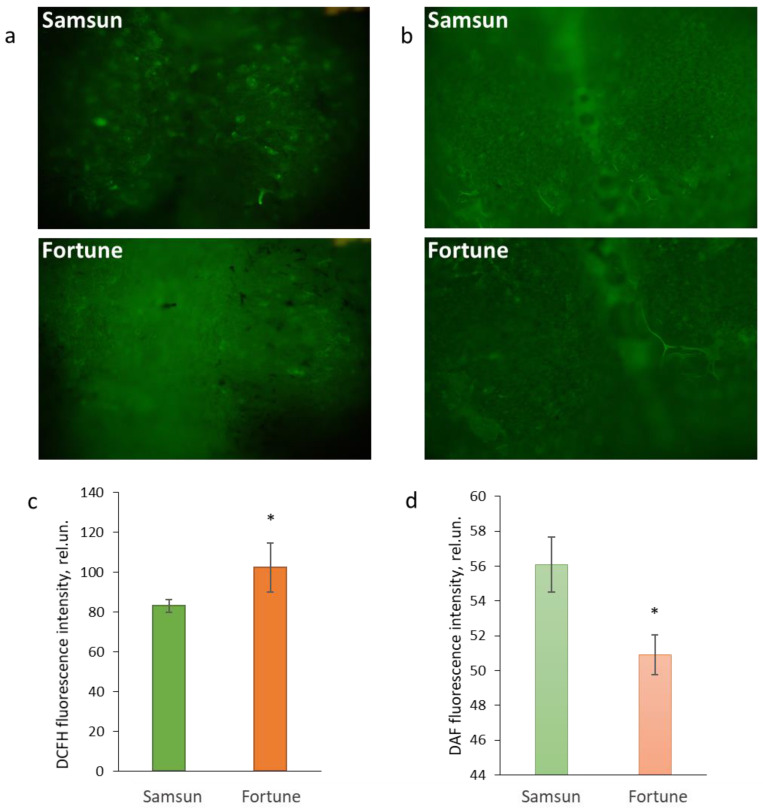
Generation of total ROS (**a**,**c**) and NO (**b**,**d**), assessed using fluorescent staining of the stigmas of tobacco plants of the Samsun and Fortune lines at the stage of maximum fertility, revealed by DCFH and DAF dyes, respectively: (**a**,**b**)—characteristic images, (**c**,**d**)—quantitative assessment of fluorescence intensity, averaged over several experiments; * indicates significant differences between the lines by the Mann–Whitney test (*p* < 0.05).

In order to assess the contribution of each component to the total oxidizing capacity, we stained fertile stigmas for extracellular ROS and nitric oxide. It turned out that the total amount of ROS (Figure 4a,b) in the F plants is significantly higher, and the amount of nitrogen oxide (Figure 4c,d) is less than in the S plants. Thus, the stigmas of plants of the two lines have a different balance of ROS and NO. This balance appears to be important for the reproductive potential of the plants. Redox homeostasis enzymes are responsible for the removal and interconversion of ROS.

### 3.3. Zymographic Assessment of SOD, APX, and CAT Activity in the Stigma Tissues

It has previously been shown that, in tobacco and other plants, the activity of redox homeostasis enzymes increases and decreases at different stages of flower development. To track these dynamics, we analyzed the stages close to the maximum stigma receptivity (=stage 3, Figure 1b). Stage 1 corresponds to loosely closed flowers that have reached their final size with a slightly colored corolla; stage 2 corresponds to flowers shortly before opening, with a partially open corolla; stage 3 corresponds to flowers after anther dehiscence with a fully open corolla; stage 4 refers to flowers the day after pollination, i.e., after the loss of stigma receptivity.

Enzyme activity was detected in gels using a zymographic method. The activities of superoxide dismutase (SOD), catalase (CAT), and ascorbate peroxidase (APX) were assessed, since hydrogen peroxide conversion is of greatest interest for tobacco. The first enzyme that converts superoxide anion radicals into hydrogen peroxide is SOD. The second stage involves the degradation of H_2_O_2_, and the main enzymes involved are APX and CAT.

**Figure 5:**
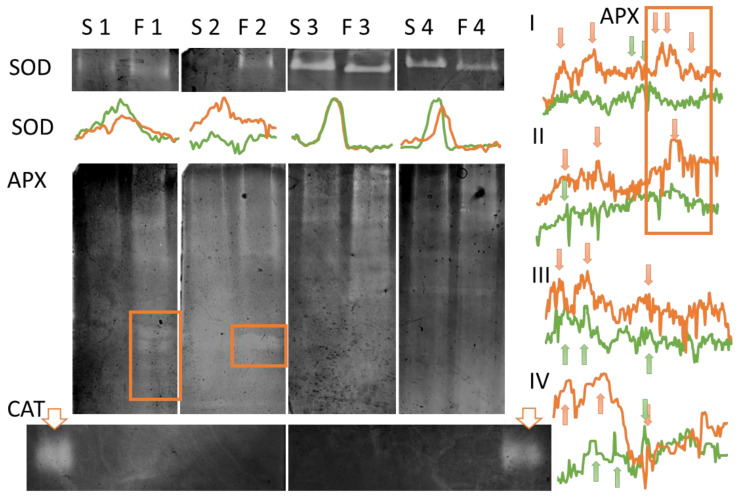
Zymographic determination of SOD, ascorbate peroxidase (APX), and catalase (CAT) activities in *Nicotiana tabacum* stigmas of the Samsun (S) and Fortune (F) lines. Total protein extracts from fresh stigmas were loaded into adjacent lanes. Before loading, the protein concentration in all extracts was determined to adjust the application dose. The number next to the lane designation for each lane corresponds to the flower development stage: 1—closed flowers; 2—partially open flowers; 3—stage of maximum fertility; 4—pollinated stigmas. Catalase activity is absent in the stigmas; therefore, to test the relevance of the method, leaf extract was applied to the outer lane (indicated by the arrow). For each gel, except for the one without catalase activity, a brightness profile is provided to enable a comparison of the brightness of the bands in the extracts from the stigmas of the two lines: Samsun—green line; Fortune—orange line; for ascorbate peroxidase, the bands are indicated by arrows of the corresponding color, the stage of maturity of the stigma is indicated above the profiles on the left. The analyzed bands, reflecting the enzymatic activity of the emerging isoforms, are highlighted with a frame on the gel.

In the dynamics line, SOD activity increased as the fertile stage approached and decreased after pollination. SOD activity differed between the two lines at stages 2 (F > S), 1, and 4 (S > F). At the fertile stage (3), activity levels did not differ (Figure 5, top panel). A single isoform was detected in all cases; in the F plants, it was slightly lighter in molecular weight (located lower in the lane). APX activity was represented by a large number of isoforms, most of which were similar in molecular weight in the two lines (Figure 5, middle panel). At all stages the total activity of the F plants was higher than that of the S plants. However, an interesting feature of the F plants was the presence of additional low-molecular-weight APX isoforms during pistil development at stages 1 and 2 (highlighted by the box). The S plants did not have such bands at any stage, nor were they present in fertile and pollinated F plant stigmas.

Catalase activity was absent in the stigmas (Figure 5, lower panel). To verify the staining method, the signal from a lane containing leaf homogenate from the same plant is shown next to the lanes containing stigma proteins.

## 4. Discussion

In this study, we test the hypothesis about the relationship between the redox balance on the stigma and the reproductive potential of tobacco plants as the most fully studied species in reproductive terms with a wet stigma. We examined two lines: Samsun, which has economic value as a source of tobacco raw material but has low pollen germination in vivo and in vitro, small fruit, and few seeds [18], and Fortune, whose plants reproduce successfully. We showed that Fortune has larger leaves, flowers, and fruit during controlled self-pollination (Figure 1). At the same time, we found a lower level of total oxidative capacity of stigma exudate and a weaker NO signal, with a moderate increase in ROS levels on the stigma (Figure 4). The question then is, how might reproductive success be related to the balance of ROS and NO on the stigma?

Pollen sensitivity to NO has been established for various plant species [37,38]. Gymnosperms do not exhibit a negative response to NO; the nitric oxide donor has a positive effect on pollen tube growth in *Pinus bungeana* in vitro [39]. Nitric oxide has a negative effect on pollen tubes of angiosperms, while pollen itself is a powerful NO generator [40]. Treatment of cucumber pollen with the nitric oxide donors GSNO and SNP inhibits both pollen germination and pollen tube growth in a dose-dependent manner [41]. The rate of pollen germination increases in the presence of the NO scavenger cPTIO. Inhibition of pollen tube growth by NO was previously shown for the lily, with the tubes making a sharp turn away from the NO source when the substance was delivered from a pipette [42]. It is suggested that intracellular NO generated in pollen grains before pollen tube formation may contribute to reproductive success through interaction with ROS produced by the stigma [43]. Ex vivo studies have shown that the interaction of pollen NO with stigmatic ROS may serve as a key aspect of “molecular compatibility”, ultimately facilitating fertilization [44]. Increased seed production in the Fortune line correlates with a significant reduction in NO generation by the stigmas of these plants, consistent with this hypothesis. At a later stage, after growth through the style, pollen tubes, on the contrary, respond positively to NO, which helps them find the entrance to the micropyle [37].

**Figure 6:**
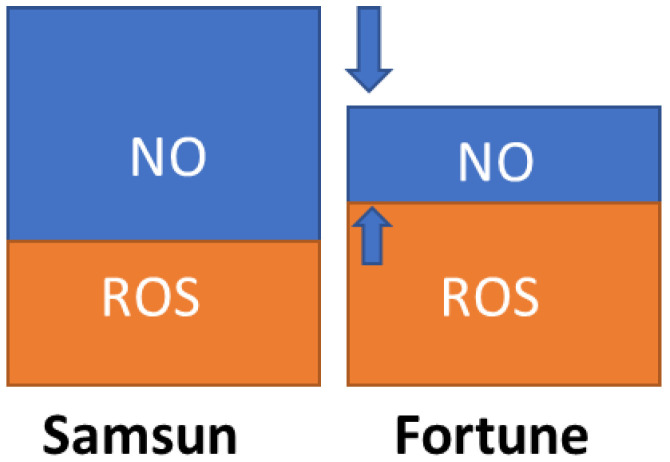
Scheme of changes in the balance of ROS and NO on the stigma of tobacco plants of the Fortune line compared to the Samsun line. The arrows indicate the decreased number of NO in the Fortune line.

The response of pollen grains and tubes to ROS has been studied both in vitro and in vivo for several species, including tobacco. It has been established that tobacco stigma exudate contains both superoxide radical and hydrogen peroxide, with the latter predominating among the ROS in the stigma of the Petit Havana variety [16]. Pollen tubes are sensitive to this exudate component; H_2_O_2_ causes hyperpolarization of the protoplast plasma membrane and, in pollen grains, it provokes accelerated synthesis of the proteins responsible for pollen germination [15]. The hypothesis that ROS balance on tobacco stigma determines the success of pollen germination was tested experimentally using an inhibitor analysis: it turned out that the addition of an SOD inhibitor to the stigma of the Petit Havana tobacco variety, which is characterized by successful reproduction under control conditions, leads to suppression of pollen germination and reduces the number of seeds [16]. Later, the enzymatic activity in the stigma tissue of this same cultivar was studied using a zymographic method [45]. It was found that the plant has several SOD isoforms, and blocking one only partially suppresses hydrogen peroxide formation. However, even a moderate reduction in H_2_O_2_ production apparently negatively impacts the reproductive process. Based on these data obtained on another variety of tobacco, it can be assumed that increased ROS levels in the stigma of the Fortune line during the stage of maximum fertility (Figure 6) may be at least one of the factors enhancing its reproductive success. Presumably, regulation of the ROS levels is associated with a successful strategy for overcoming pollen damage during megasporogenesis [46], or neutralizing excess reactive oxygen species [47,48], stabilizing the processes of tapetum and pollen formation. These regulatory processes involve oxalates, which are abundant in specialized anther-opening cells throughout the *Solanaceae* family [29,30], and in particular in tobacco [49].

The Fortune plants exhibit interesting dynamics of redox homeostasis enzymes: at stage 2, preceding fertility, SOD activity in their stigmas is higher than in the Samsun plants, and additional APX isoforms are detected at stages 1 and 2 (Figure 5). This may be related to ongoing growth: all floral organs are larger in the Fortune line than in the Samsun line (Figure 1). The involvement of ROS in the development of reproductive organs is one of the hypotheses explaining why ROS appear on the stigma when pollen cannot yet reach it [44]. In his comprehensive 1984 review of peroxidases in the pistil, Bredemeijer wrote that most pistil peroxidases are involved in the processes associated with growth, development, and aging. In his opinion, only tissue-specific peroxidases in the vascular tissue of the style can play a direct role in regulating pollen tube growth [50]. Much later, tissue-specific peroxidases SSP (stigma-specific peroxidase) were first described in the stigmas of *Senecio squalidus* L. (*Asteraceae*) [51], and their co-localization with stigmatic ROS was demonstrated at the fertile stage [43].

At stage 3, SOD activity is equal in the stigmas of the Samsun and Fortune plants; the level of total oxidative capacity is higher in the former, and the level of ROS is higher in the latter (Figure 4a,b). Such enzyme dynamics indicate a complex regulation of the balance between ROS and NO during flower development, which is consistent with the data obtained on plants with different stigma types (dry, semi-dry, and wet), such as sunflower [52,53], olive [54], and *Senecio squalidus* [43].

Thus, regulation of ROS in generative organs is important for successful reproductive function [3,47]. Harmonious changes in osmotic processes and antioxidant enzyme activity can significantly influence another development, pollen formation, pollen germination on a wet stigma, and pollen filament growth, which requires significant modification of the structure and metabolism of the involved cells. In some cases, these processes should induce programmed cell death after developmental disturbances due to impaired microsporogenesis [55] or during pollen germination in the case of self-incompatibility due to cytoskeletal disruption caused by caspase action [56,57]. Similarly, cell death associated with ROS production occurs during endoreduplication, for example, in tapetum cells or inner stigma cells that increase ploidy for the superproduction of exine [48,55,58] and mucus, respectively [59,60]. These processes are also often directly linked to the biosynthesis of another phytoregulator, ethylene [61].

## 5. Conclusions

The results obtained in this work demonstrate the importance of monitoring the development indicators of flowering organs, controlling the processes of the formation of fertile pollen, even in the case of its obvious excess amount, as a basis for ensuring not only the reliability of generative development but productivity.

## Data Availability

The original contributions presented in this study are included in the article/Supplementary Materials. Further inquiries can be directed to the corresponding authors.

## Supplementary Materials

The following supporting information can be downloaded at https://www.mdpi.com/article/10.3390/cimb48050432/s1.
