# Supplementary material for: ROS Generation and Redox Enzyme Activity in the Stigmas of Two Tobacco Plant Lines with Different Seed Productivity Levels
Source: Curr Issues Mol Biol. 2026 Apr 22;48(5):432. doi: 10.3390/cimb48050432 (PMC13204609; doi:10.3390/cimb48050432)
Supplement: Supplementary file 1 [file cimb-48-00432-s001.zip › cimb-4232004-supplementary.pdf]

Supplement

Original images

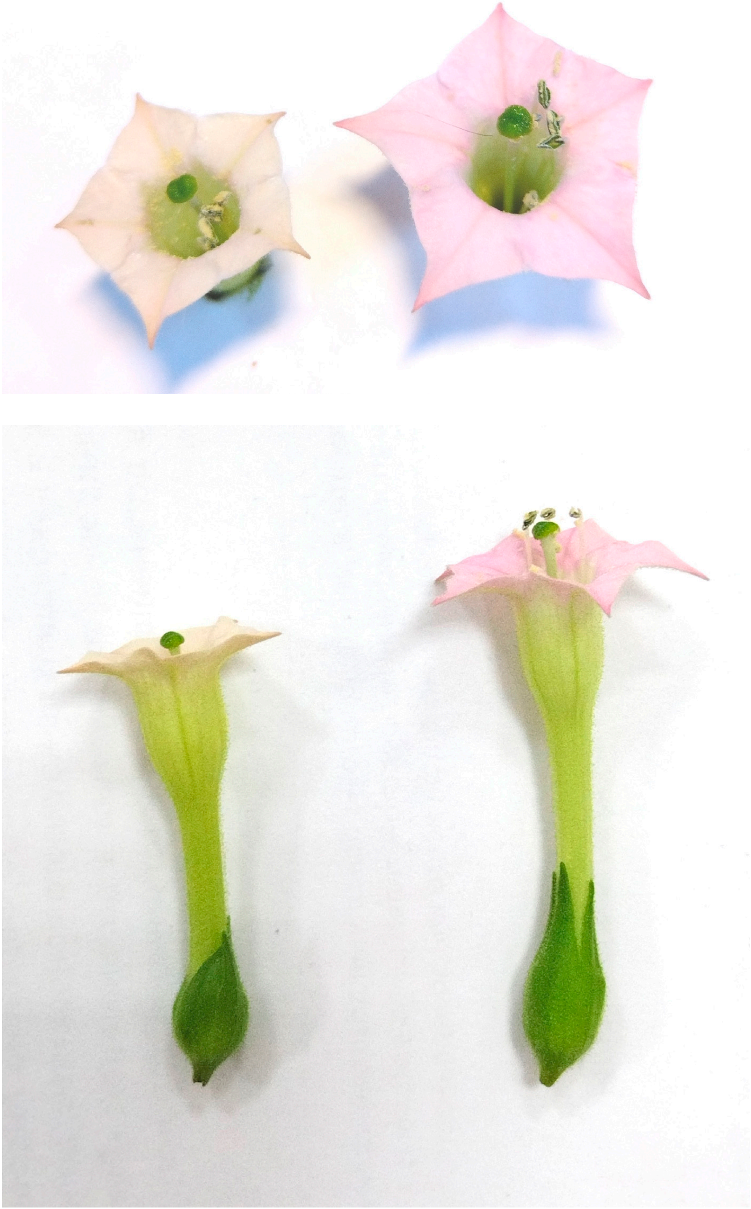

Figure S1. A comparison of tobacco flowers from two lines: Samsun (left) and Fortuna (right). The differences lie in the uneven position of the anthers and stigma.

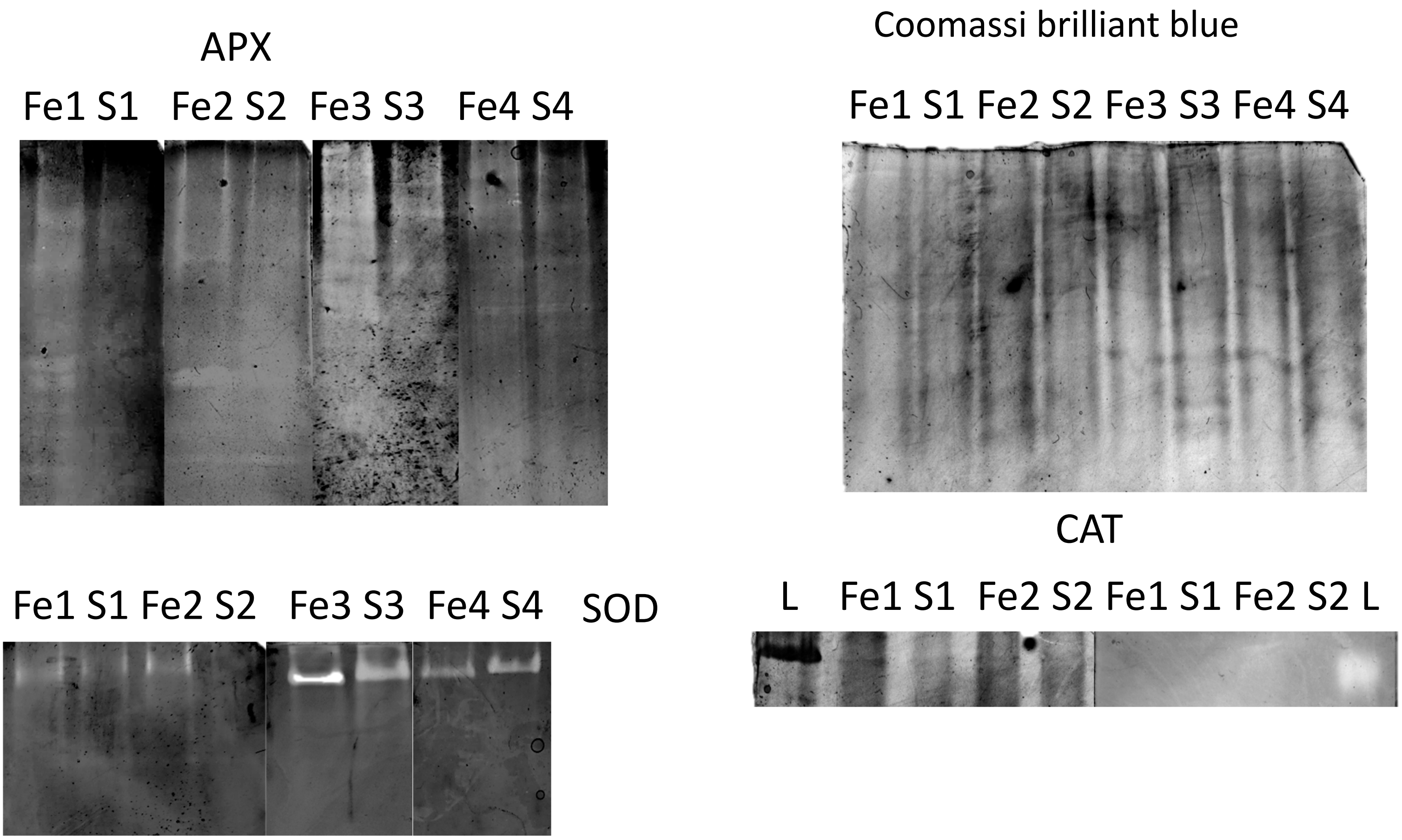

Figure S2. Zymographic determination of SOD, ascorbate peroxidase (APX), and catalase (CAT) activities in *Nicotiana tabacum* stigmas
